# A cross-sectional study of salivary and gut microbiomes in hemodialysis patients with heart failure with preserved ejection fraction

**DOI:** 10.3389/fcimb.2025.1683657

**Published:** 2026-01-14

**Authors:** Lu Li, Qiaona Zhang, Lingge Zhang, Ru Wei, Yunlong Qin, Jin Zhao, Hao Wu

**Affiliations:** 1 Department of Nephrology, First Affiliated Hospital of Xi’an Medical University, Xi’an Medical University, Xi’an, China; 2 Department of Nephrology, Bethune International Peace Hospital, Shijiazhuang, China; 3 Department of Nephrology, Xijing Hospital, Fourth Military Medical University, Xi’an, China

**Keywords:** dysbiosis, gut microbiota, heart failure, hemodialysis, oral microbiota

## Abstract

**Background:**

Heart failure (HF) is a primary cause of death in patients on maintenance hemodialysis (MHD), yet the role of microbial dysbiosis is poorly defined. This study characterized the salivary and gut microbiomes of MHD patients with heart failure with preserved ejection fraction (HFpEF), those without HF (NHF), and healthy controls (CON).

**Methods:**

In this cross-sectional study (n=88), we compared the salivary and fecal microbiomes of HFpEF (n=30), NHF (n=30), and CON (n=28) groups using 16S rRNA gene sequencing. Microbial community structure and composition were analyzed.

**Results:**

Alpha diversity and Beta diversity analysis revealed a distinct salivary microbial structure, which effectively distinguished the MHD group from the Con group (P < 0.05). Conversely, the overall gut community structure showed no significant separation. At the genus level, both MHD groups showed depletion of salivary Veillonella and gut Faecalibacterium compared to controls. Notably, LEfSe analysis highlighted salivary Anaerocolumna as a promising candidate feature associated with the HFpEF group.

**Conclusion:**

Our analyses suggest that HFpEF in MHD patients may be associated with structural alterations in the oral microbiome, which appear more pronounced than those in the gut. Specific oral microbial signatures, particularly the enrichment of Anaerocolumna, showed associations with the HFpEF cohort in our study. This preliminary evidence positions the oral microbiome as an area worthy of further investigation for its potential role in this high-risk population.

## 1 Introduction

End-stage renal disease (ESRD) has emerged as a significant global public health challenge. According to data from the International Society of Nephrology (ISN), approximately 850 million adults worldwide are affected by chronic kidney disease, with a proportion of these patients progressing to ESRD, necessitating renal replacement therapy, including dialysis and kidney transplantation, to sustain life (Jha et al., 2013). Whilst dialysis treatment has notably extended patient survival, the mortality rate among dialysis patients remains regrettably high, with heart failure (HF) being one of its primary fatal complications. The US Renal Data System (Collins et al., 2012) reports an annual mortality rate of around 20% for dialysis patients, and intriguingly, heart failure accounts for 30% to 50% of these deaths, making it a leading cause (Collins et al., 2012). Of particular concern, heart failure with preserved ejection fraction (HFpEF) is highly prevalent in this population. The pathogenesis of HF is complex, involving a myriad of factors such as immune dysfunction, metabolic abnormalities, and chronic inflammation (Jia et al., 2019). Presently, the high incidence of HF, particularly HFpEF, in dialysis patients represents a critical issue, profoundly impacting both their quality of life and overall prognosis.

In recent years, the role of the microbiome in kidney and cardiovascular diseases has garnered increasing attention, particularly the demonstrable link between gut microbiome dysbiosis and a range of pathological states. An imbalance in the gut microbiome can influence host immune responses, metabolism, and inflammatory pathways, thereby contributing to the development and progression of HF (Tang et al., 2019; Ren et al., 2024). Recent studies have highlighted the complex interactions between gut microbiota, diet, and microbial metabolites in chronic heart failure (Fadhillah et al., 2025). Within the dialysis patient population, gut microbiota dysregulation may exacerbate chronic inflammation and is associated with immune system suppression, which can further compound complications such as HF (Luo et al., 2021). Nevertheless, recent research into the salivary microbiome has similarly unveiled its potential involvement in systemic diseases. The microbial communities found in saliva are intrinsically linked to oral health, yet also demonstrate potential connections to broader systemic conditions like diabetes and rheumatoid arthritis (Sedghi et al., 2021; Hou et al., 2022). Furthermore, studies suggest that the composition of the salivary microbiome may interact with gut microbial function, influencing host immune responses and metabolic status (Moreno et al., 2023; Kis-György et al., 2024).

Although the salivary microbiome’s role in various systemic diseases has received some recognition, a systematic investigation into the interrelationship between salivary and gut microbiomes in maintenance dialysis patients remains largely underexplored, particularly concerning changes observed in those with HFpEF. Existing research indicates that the salivary microbiome plays a significant part in the context of diabetes, infections, and other complications experienced by dialysis patients (Duan et al., 2020; Liu et al., 2022). However, current studies often focus on a single microbial community or localized conditions, lacking the comprehensive, multi-sample, and multi-omic analyses required to fully assess the interplay between salivary and gut microbiomes and their impact on HF.

Consequently, this study aims to employ 16S rRNA gene high-throughput sequencing to systematically compare the salivary and fecal microbiome compositions of maintenance haemodialysis patients with HFpEF, those without HF, and healthy control subjects. Our objective is to uncover the specific impact of HFpEF on the salivary and gut microbiomes of dialysis patients, to further elucidate the intricate mechanisms of interaction between these two microbial communities, and ultimately, to provide a robust theoretical basis for the future development of microbiome-based biomarkers or therapeutic strategies.

## 2 Materials and methods

### 2.1 Ethics statement

This observational study was registered in the Chinese Clinical Trial Registry (ChicCTR2400079717). All experiments involving human participants were conducted in accordance with the ethical policies and procedures approved by the Institutional Review and Ethics Board of the First Affiliated Hospital, Xi’an Medical University (XYYFY2022LSKY-054-01). All participants provided written informed consent prior to enrollment.

### 2.2 Recruitment and sample collection

This study employed a cross-sectional design. We first identified 30 maintenance hemodialysis patients with HFpEF group who met the inclusion criteria at the dialysis center of the First Affiliated Hospital of Xi’an Medical University.

To control for confounding bias, we performed 1:1 propensity score matching without replacement to select controls (the NHF group) for the HFpEF group from a larger cohort of maintenance hemodialysis patients without heart failure. Propensity scores were estimated using a logistic regression model incorporating age, sex, and dialysis vintage, with matching conducted via the greedy nearest neighbor algorithm under a caliper width of 0.2 times the standard deviation of the logit propensity score. Potential controls were randomly sorted prior to matching. This process yielded a well-balanced NHF group of 30 participants. Additionally, 30 healthy controls (CON group) broadly matched on age and sex were recruited. Demographic data, clinical parameters, medication history, and the primary cause of ESRD for all patients were collected from electronic medical records or questionnaires.

Inclusion criteria for the HF group were: 1) age ≥ 18 years; 2) regular hemodialysis treatment (3 times per week for > 4 hours per session) for ≥ 1 year; 3) a diagnosis of heart failure, specifically HFpEF, confirmed by a cardiologist according to the Chinese Guidelines for the Diagnosis and Treatment of Heart Failure (2018); and 4) provision of signed informed consent.

Inclusion criteria for the NHF group were: 1) age ≥ 18 years; 2) regular hemodialysis treatment (3 times per week for > 4 hours per session) for ≥ 1 year; 3) no current or historical diagnosis of heart failure; and 4) provision of signed informed consent.

Inclusion criteria for the healthy control group were: 1) age ≥ 18 years; 2) no history of chronic kidney disease or any form of renal replacement therapy; 3) no diagnosis of heart failure or other major cardiovascular diseases; 4) no history of gastrointestinal diseases, autoimmune disorders, or chronic inflammatory conditions; and 5) provision of signed informed consent.

Exclusion criteria for all groups were: 1) history of renal transplantation; 2) pregnancy; 3) Co-existing Diabetes Mellitus (Type 1 or Type 2); 4) Presence of other major systemic diseases that could significantly influence systemic inflammation or the microbiome; 5) Use of antibiotics, corticosteroids, immunosuppressants, probiotics, or prebiotics within the past 3 months; 6) History of acute infectious diseases in the past 3 months; 7) Presence of severe oral conditions, including active severe periodontitis, multiple untreated dental caries, or oral mucosal disease;8) Received systemic periodontal treatment in the past year; 9) Inability to provide informed consent.

Gut samples were collected from a clinical cohort consisting of either hospitalized patients or MHD patients during their routine sessions in our hospital-based dialysis unit. This design ensured that all samples were procured and processed under direct clinical supervision. Immediately following defecation, a research staff member received the fresh sample. Using sterile utensils, a representative aliquot was promptly transferred into a cryogenic vial. The vial was then immediately transferred to a -80°C freezer located within the same clinical unit. The entire process, from collection to permanent storage at -80°C, was consistently completed within 2–3 minutes for all samples. All gut samples were transported to the sequencing facility for 16S rRNA gene sequencing within 4 weeks of collection.

Oral samples (unstimulated whole saliva) were collected between 7:00 AM and 9:00 AM after an overnight fast. Subjects were instructed to allow saliva to naturally accumulate and then spit into a sterile specimen tube for at least 1 minute, aiming for a volume of approximately 1–2 mL. Collected saliva was transferred to an ultra-low temperature refrigerator at -80°C within 2 hours.

Due to issues with DNA concentration or sequencing quality in some samples, a total of 88 saliva samples (30 from the NHF group, 30 from the HFpEF group, and 28 from the healthy control group) and 88 gut samples (30 from the NHF group, 30 from the HF group, and 28 from the healthy control group) were successfully sequenced and included in the final analysis.

### 2.3 DNA extraction and 16S rRNA gene amplification

Total genomic DNA was extracted from all saliva and gut samples using the DNeasy PowerSoil Pro Kit (Qiagen, Hilden, Germany) following the manufacturer’s instructions.

The V3-V4 hypervariable regions of the 16S rRNA gene were amplified by PCR using specific primers 341F (5’-CCTACGGGNGGCWGCAG-3’) (forward) and 806R (5’-GACTACHVGGGTATCTAAT-3’) (reverse). PCR reactions were performed in triplicate for each sample, with a total volume of 25 µL containing 12.5 µL 2X KAPA HiFi HotStart ReadyMix, 2.5 µL of each primer (10 µM), and 5 µL template DNA. The thermal cycling conditions were as follows: initial denaturation at 95°C for 3 min; 25 cycles of 95°C for 30 sec (denaturation), 55°C for 30 sec (annealing), and 72°C for 30 sec (extension); followed by a final extension at 72°C for 5 min. PCR products were purified using Agencourt AMPure XP beads (Beckman Coulter, Brea, CA, USA) and quantified using a Qubit 2.0 Fluorometer (Life Technologies, Carlsbad, CA, USA).

### 2.4 Microbiota sequencing

Purified and quantified 16S rRNA gene PCR products were pooled in equimolar concentrations and subjected to paired-end sequencing (2x250 bp) on an Illumina MiSeq platform (Illumina, San Diego, CA, USA) at Origingene (Shanghai, China).

### 2.5 Functional prediction analysis

Functional prediction was performed using PICRUSt (version 1.1.4) to infer metagenomic functions from the 16S rRNA gene sequencing data. ASV representative sequences were placed into a reference phylogenetic tree, and hidden-state prediction of gene families was conducted using the castor algorithm. The predicted gene families were annotated to KEGG Orthologs (KOs) and COG categories, and further mapped to KEGG pathways and EC numbers. Statistical analysis of functional differences between groups was performed using the STAMP software package. G-test (for large sample sizes with >20 annotated functional genes) or Fisher’s exact test (for small sample sizes with ≤20 annotated functional genes) was applied for pairwise comparisons, with a significance threshold of P < 0.05.

### 2.6 Statistical analysis

Raw sequencing reads were processed using the DADA2 pipeline (via QIIME2 2024.2) to resolve amplicon sequence variants (ASVs). Briefly, sequence reads were quality-filtered, denoised, merged, and chimeras were removed. Taxonomic assignment of the resulting ASVs was performed against the SILVA database (release 132) using a trained classifier.

Alpha diversity indices (Chao1, Shannon, and Simpson) were calculated using Mothur version 1.41.0. Differences in alpha-diversity among the three groups were assessed using Kruskal-Wallis tests. For significant Kruskal-Wallis results, *post-hoc* pairwise comparisons were performed using Dunn’s test with Benjamini-Hochberg adjustment for multiple comparisons.

Beta diversity was visualized using non-metric multidimensional scaling (NMDS) plots based on Bray-Curtis dissimilarity matrices, generated using R statistical software (version 4.2.1) with the vegan package. The statistical significance of differences in microbial community structure among the three groups was assessed using PERMANOVA (Permutational Multivariate Analysis of Variance) with 999 permutations. For significant PERMANOVA results, pairwise PERMANOVA tests were conducted with Bonferroni correction for multiple comparisons.

Differential abundance analysis of microbial taxa among the three groups was performed using Kruskal-Wallis tests. P-values were adjusted for multiple comparisons using the Benjamini-Hochberg method to control the false discovery rate (FDR). For specific taxa showing significance in the Kruskal-Wallis test, *post-hoc* pairwise comparisons were conducted using Wilcoxon rank-sum tests with Benjamini-Hochberg adjustment.

Baseline demographic and clinical characteristics were compared among the three groups using appropriate statistical tests: ANOVA or Kruskal-Wallis tests for continuous variables, and Chi-square tests or Fisher’s exact tests for categorical variables. For significant omnibus test results (ANOVA/Kruskal-Wallis) for continuous variables, *post-hoc* pairwise comparisons were performed.

Statistical significance was defined as an FDR-adjusted P-value (q-value) of less than 0.05. All statistical analyses were performed using R statistical software (version 4.2.1).

## 3 Result

### 3.1 Clinical characteristics of study participants

A total of 88 participants were enrolled in this study, comprising 30 maintenance hemodialysis patients with HFpEF group, 30 maintenance hemodialysis patients without HF (NHF group), and 28 healthy control subjects (CON group). The baseline demographic and clinical characteristics of all participants are summarized in **Table 1**.

**Table 1:** Balance diagnostics after propensity score matching between HFpEF and NHF groups.

Variable	HFpEF (n=30)	NHF (n=30)	Standardized mean difference (SMD)
Age (years), Mean (SD)	55.4 (11.3)	50.2 (15.6)	0.37
Female, n (%)	12 (40.0%)	13 (43.3%)	0.07
Dialysis Vintage (months), Mean (SD)	45.2 (18.7)	42.1 (16.3)	0.17
Additional covariates			
BMI (kg/m²), Mean (SD)	22.1 (4.1)	23.5 (4.5)	0.33
- Hypertension	24 (80.0%)	22 (73.3%)	0.16
- Diabetes Mellitus	14 (46.7%)	11 (36.7%)	0.2
- Hemoglobin (g/L), Mean (SD)	104.3 (22.1)	107.6 (25.8)	0.14
- Albumin (g/L), Mean (SD)	36.9 (4.3)	37.5 (6.6)	0.11
- Sodium (mmol/L), Mean (SD)	136.8 (4.2)	137.8 (4.5)	0.23
- Potassium (mmol/L), Mean (SD)	4.5 (0.8)	4.4 (0.7)	0.14
- Calcium (mmol/L), Mean (SD)	2.06 (0.31)	2.10 (0.24)	0.15
- Phosphorus (mmol/L), Mean (SD)	1.77 (0.60)	1.75 (0.41)	0.04
- PTH (pg/mL), Median (IQR)	288.9 [175-533]	354.4 [121-566]	0.22
- Systolic BP (mmHg), Mean (SD)	142.6 (24.3)	143.8 (22.7)	0.05
- BNP (pg/mL), Median (IQR)	21547 [15892-25000]	2645 [426-5634]	2.47
- Ejection fraction (%), Mean (SD)	53.5 (8.2)	58.7 (5.3)	0.75

As shown in **Table 1**, there were no significant differences in age, female sex, height, or diastolic blood pressure among the three groups. However, significant differences were observed in systolic blood pressure. Consistent with their respective conditions, the HF group exhibited significantly higher BNP levels and lower ejection fraction compared to both NHF and CON groups. Both the hemodialysis groups (HEpEF and NHF) showed expected significant differences in renal function markers (Urea, Creatinine), Hemoglobin, Sodium, and Potassium levels when compared to the healthy control group.

### 3.2 Alpha diversity of oral and gut microbiota

**Figure 1:**
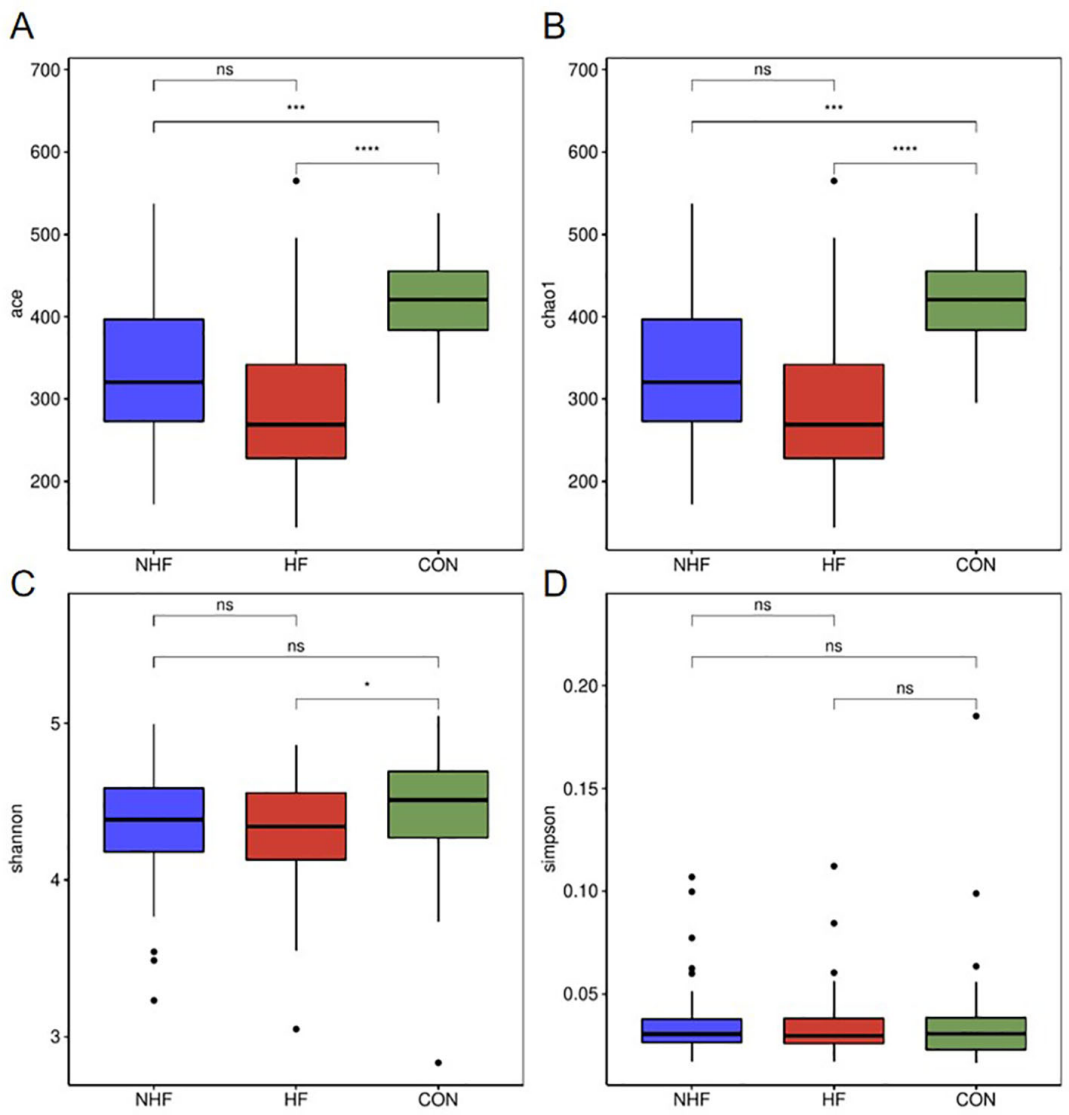
The alpha diversity indices of oral microbiot. **(A)** ACE index; **(B)** Chao1 index; **(C)** Shannon index; **(D)** Simpson index. ns P>0.05, *P<0.05, ***P<0.001, ****P<0.0001.

Alpha diversity of both oral and gut microbiota was assessed across the NHF, HFpEF, and CON groups using a panel of ecological indices. For the oral microbiota (**Figure 1**), compared with the CON group, the ACE and the Chao1 index in HF group (****P < 0.0001) and NHF group (***P < 0.001) were significantly decreased. Similarly, the Shannon index, which reflects community evenness and richness, was also significantly elevated in the CON group relative to the HF group (P < 0.05). No significant differences in these indices were observed between the NHF group and the HF group. In contrast, the Simpson index did not differ significantly among all groups.

**Figure 2:**
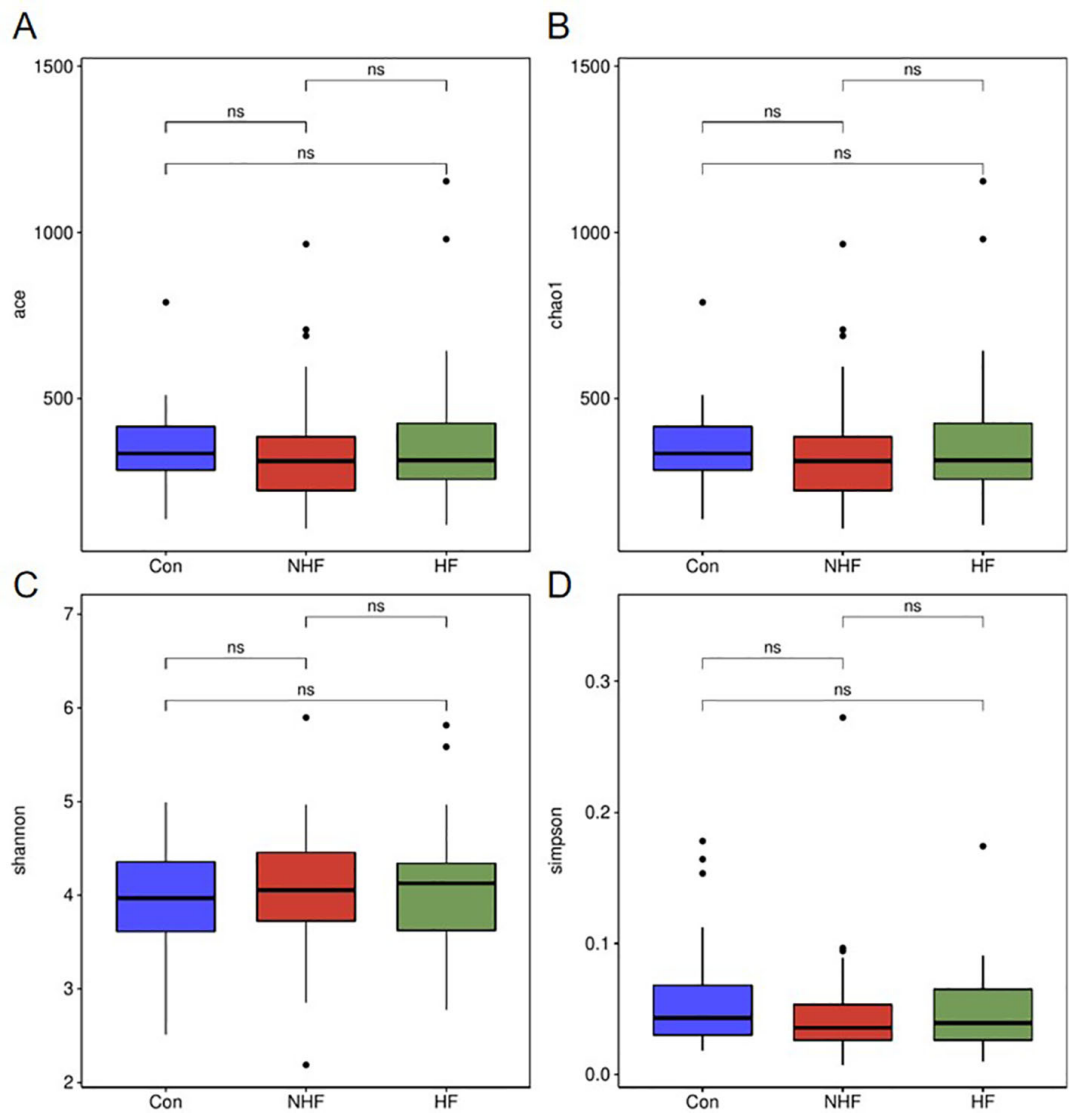
The alpha diversity indices of gut microbiot. **(A)** ACE index; **(B)** Chao1 index; **(C)** Shannon index; **(D)** Simpson index. ns P>0.05.

For the gut microbiota (**Figure 2**), no significant differences in alpha diversity were detected among the three groups (P > 0.05). All evaluated diversity indices remained statistically similar across the NHF, HFpEF, and CON cohorts.

### 3.3 Beta diversity of oral and gut microbiota

**Figure 3:**
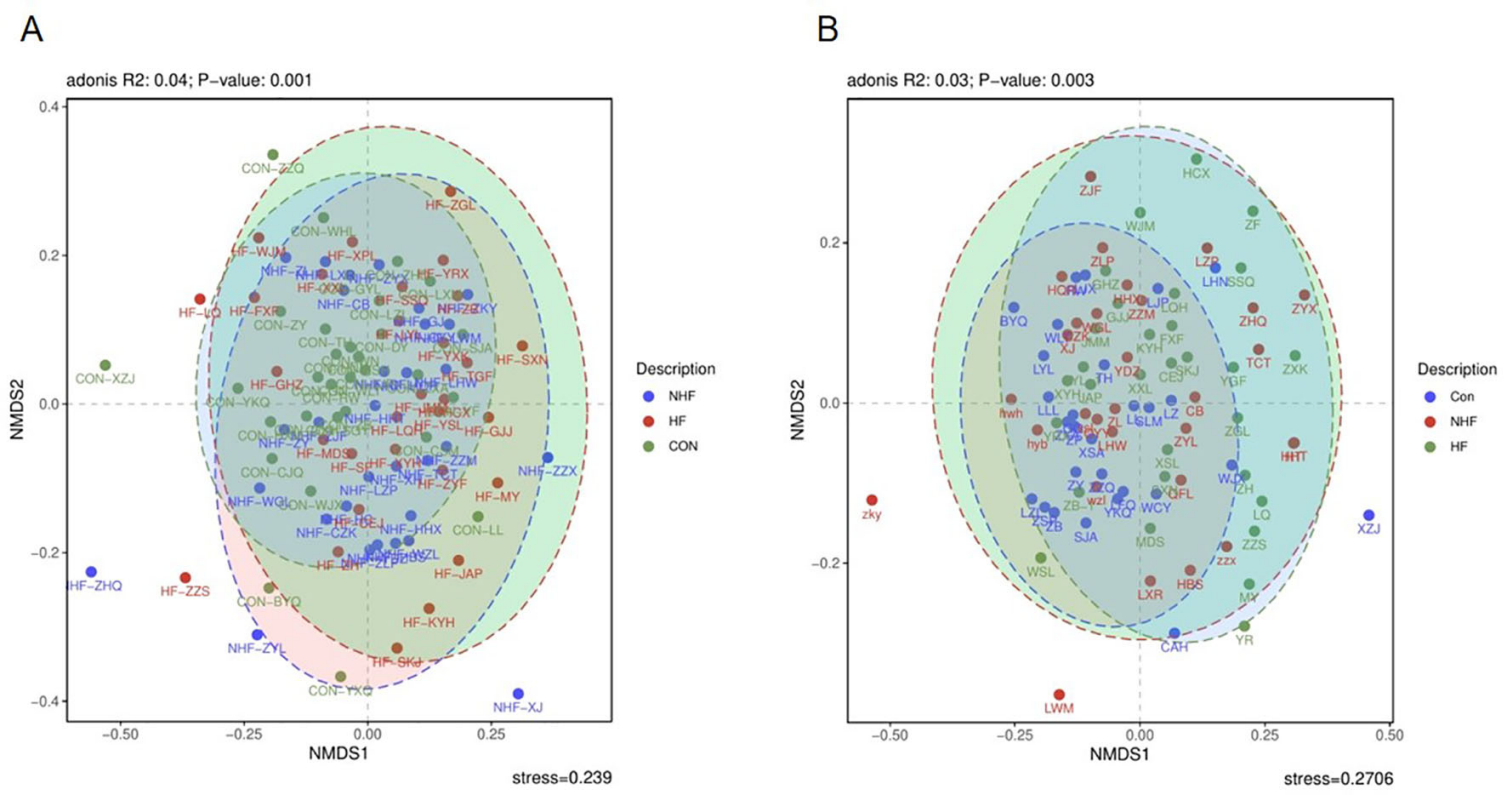
The beta diversity of all groups Differences in the composition of bacterial com munities in all groups of oral **(A)** and gut **(B)**.

For the oral microbiota (**Figure 3A**), the overall bacterial community structure showed clear differences among the three groups (Adonis R² = 0.04, P = 0.001). The healthy control group tended to differ from both hemodialysis groups, and patients with HF showed variations compared to those without HF. This indicates that both hemodialysis and HF status are associated with changes in the oral microbiota.

In contrast, the gut microbiota (**Figure 3B**), statistically significant differences were also detected (Adonis R² = 0.03, P = 0.003), but the effect size was even smaller. Visually, no clear separation in community structure was observed among the three groups, with extensive overlap in multidimensional space. These results indicate that HF status does not substantially alter gut microbiota composition in patients undergoing hemodialysis despite statistical significance.

### 3.4 Gut microbial community composition

**Figure 4:**
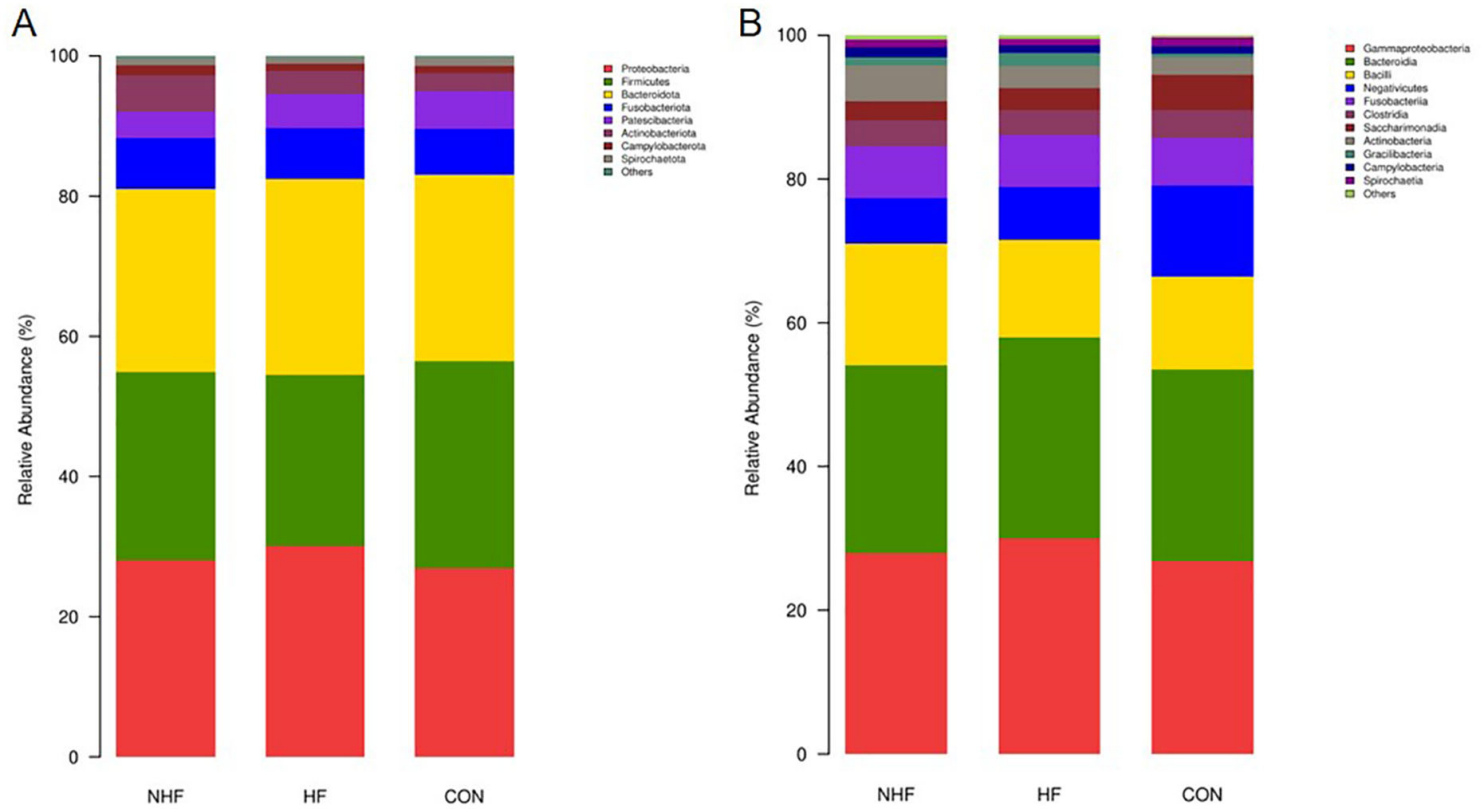
The relative abundance of oral bacterial communities at the phylum **(A)** and class **(B)** levels.

As shown in **Figure 4**, the dominant oral bacterial phylum in all groups were Proteobacteria, Bacteroidota, Firmicutes, and Fusobacteriota, with similar overall distributions (**Figure 4A**). Specifically, the abundance of Proteobacteria was significantly elevated in the HF group compared to the CON group (P < 0.05). Conversely, the abundance of Firmicutes showed a significant gradation, being highest in the CON group, intermediate in the NHF group, and lowest in the HF group (P < 0.05). No significant differences were observed for Bacteroidota and Fusobacteriota across the groups. At the class level (**Figure 4B**), within the Firmicutes phylum, Bacilli were significantly elevated in NHF, while Negativicutes were markedly enriched in CON. Beyond Firmicutes, Gammaproteobacteria were more abundant in HF, whereas Saccharimonadia dominated in CON and Actinobacteria in NHF. These results reflect a class-level taxonomic restructuring linked to group-specific physiological states.

**Figure 5:**
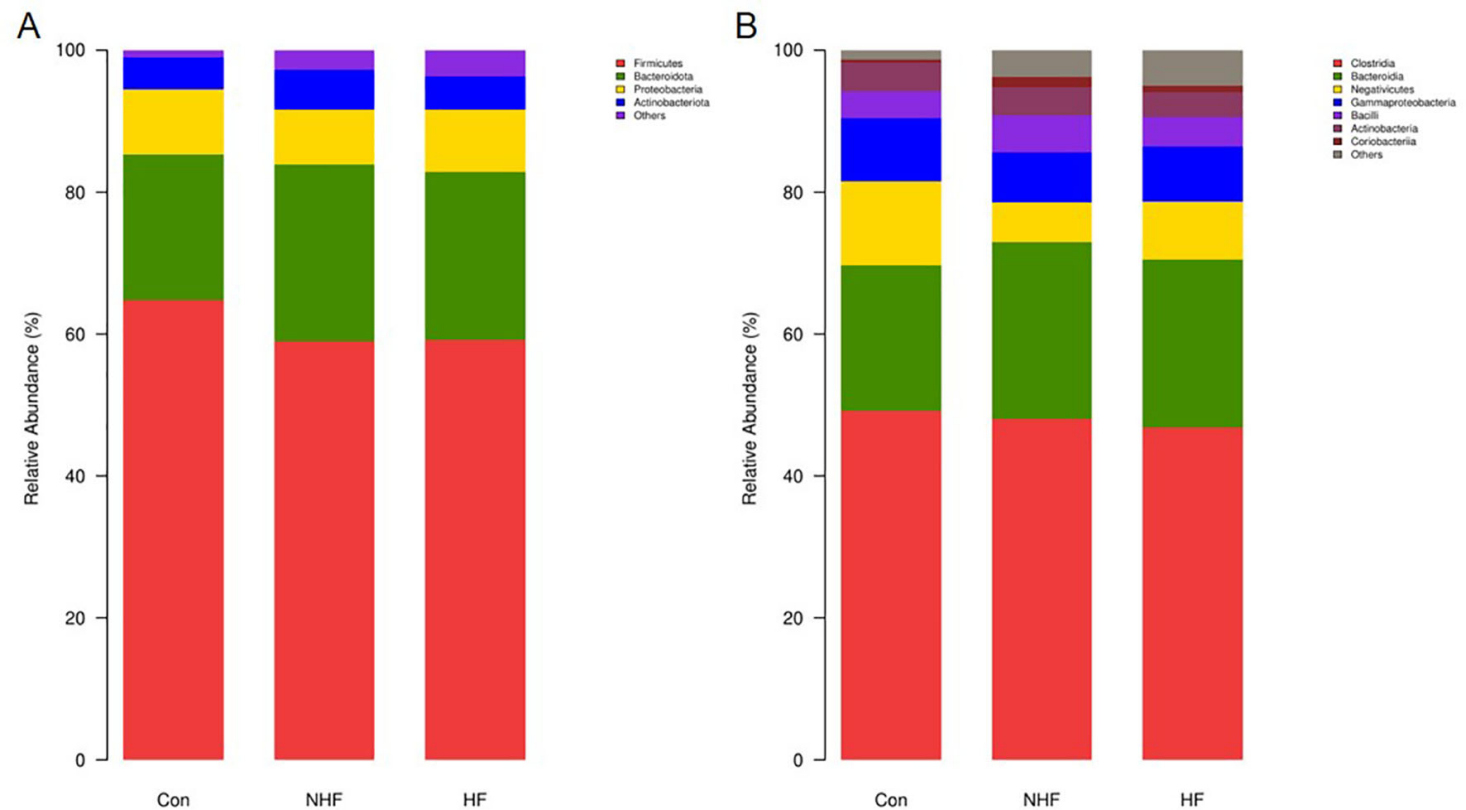
The relative abundance of gut bacterial communities at the phylum **(A)** and class **(B)** levels.

For the phylum level of gut microbiota (**Figure 5A**), Firmicutes, Bacteroidota, Proteobacteria, and Actinobacteriota were the main phyla across groups. The Firmicutes-to-Bacteroidota (F/B) ratio were significantly lower in NHF and HF groups compared to CON, driven by a decrease in Firmicutes and an increase in Bacteroidota. Additionally, Proteobacteria was lowest in NHF, while Actinobacteriota were most abundant in the same group. Analysis of gut microbiota at the class level revealed consistent shifts in the NHF and HF groups compared to CON (**Figure 5B**). The most prominent change was a marked reduction in Negativicutes. Conversely, Bacteroidia abundance increased in both condition groups. While Clostridia remained dominant across all groups, its levels were slightly lower in HF.

**Figure 6:**
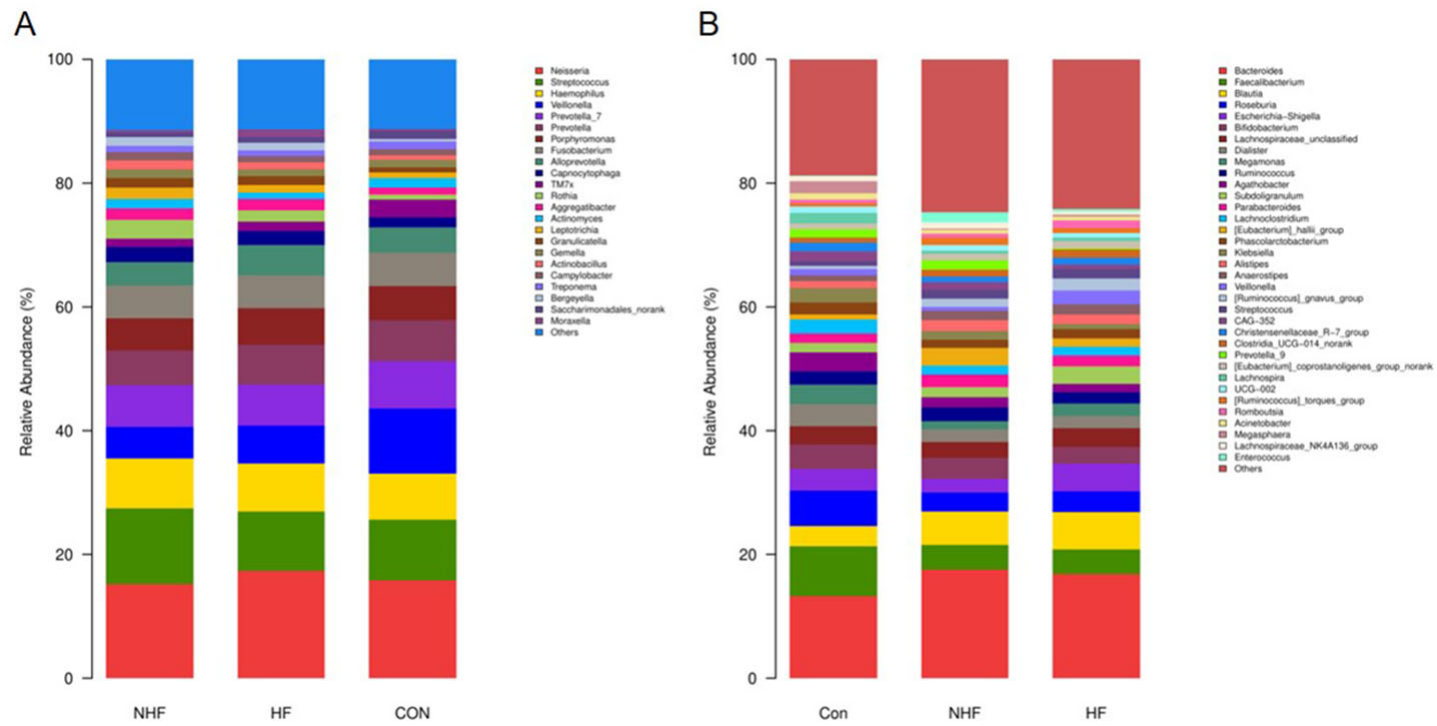
The relative abundance of oral **(A)** and gut **(B)** bacterial communities at the genus levels.

In the genus level of oral microbiota (**Figure 6A**), the CON group was characterized by a high relative abundance of Neisseria and Streptococcus. In contrast, the HF group showed a marked increase in genera such as Prevotella and Veillonella, while the NHF group exhibited an intermediate profile. In the genus level of gut microbiota (**Figure 6B**), the CON group was enriched with beneficial genera, including Faecalibacterium and Roseburia. The HF group demonstrated a notable elevation in Escherichia-Shigella and Klebsiella, alongside a reduction in several commensal butyrate-producing genera. The NHF group again displayed a transitional community structure between CON and HF.

These findings demonstrate condition-specific shifts in both oral and gut microbiota at the genus level, with the HF group showing the most pronounced dysbiotic signatures in both compartments.

### 3.5 LEfSe analysis

**Figure 7:**
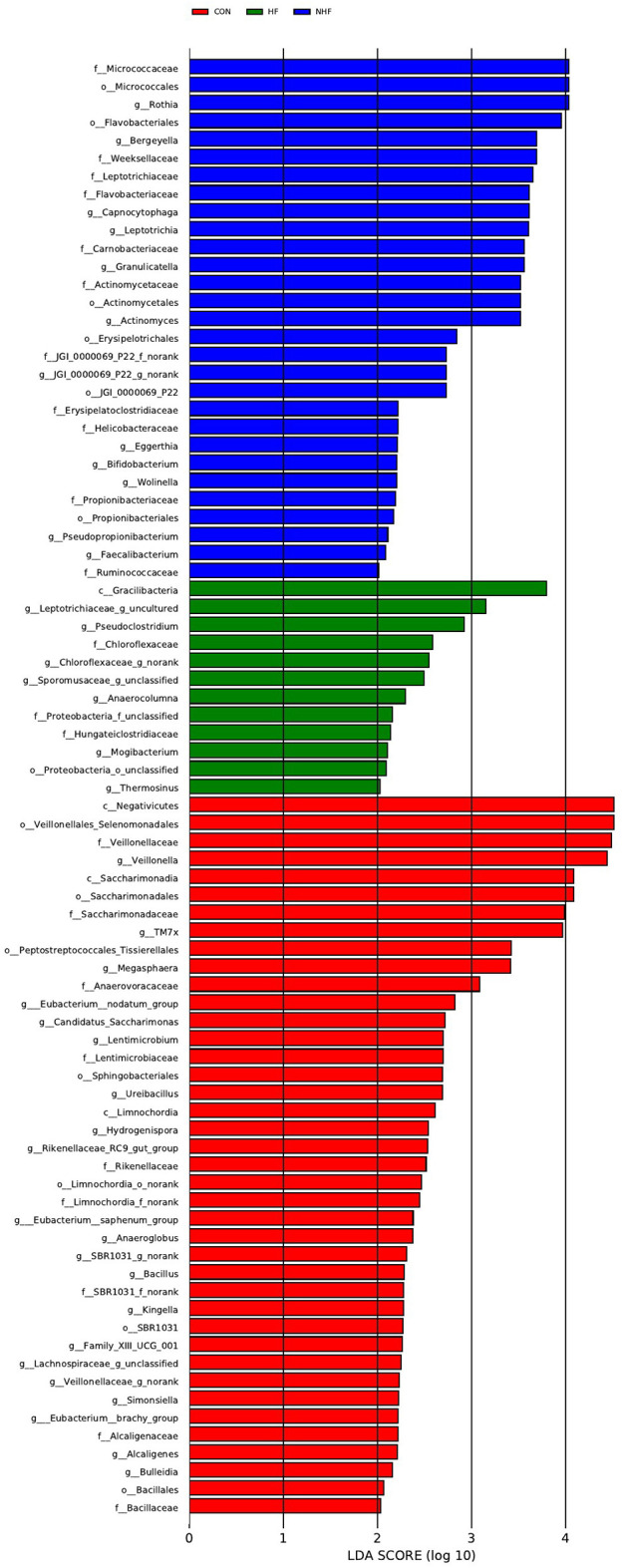
LEfSe at genus level: oral samples.

**Figure 8:**
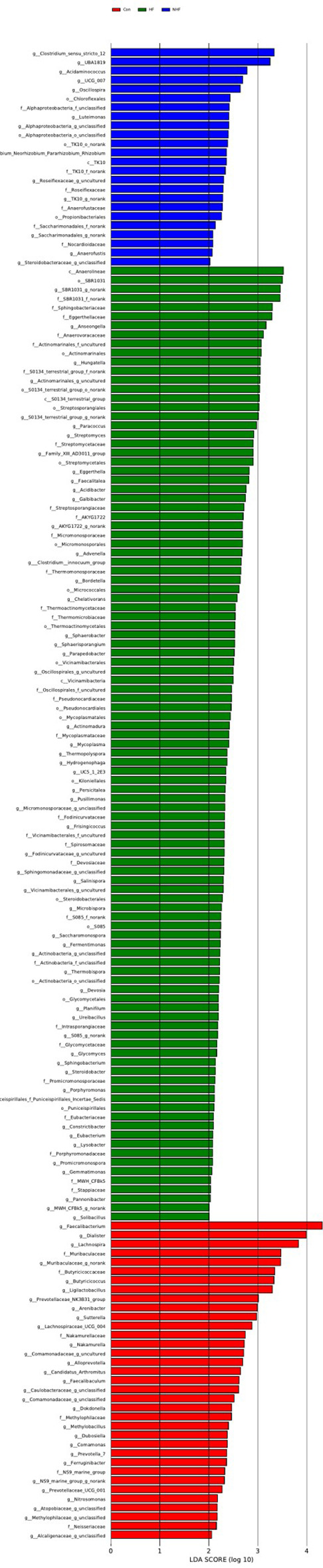
LEfSe at genus level: gut samples.

LEfSe analysis (LDA score > 2.0, P < 0.05) was conducted to investigate bacterial features associations with HF in MHD patients (**Figures 7**, **8**). In the oral samples (**Figure 7**), Anaerocolumna was enriched in the HF group, while Veillonella was enriched in the healthy controls, consistent with the relative abundance findings.

In the gut samples (**Figure 8**), multiple genera were identified: Mycoplasma and Thermospora were enriched in the HF group, whereas Faecalibacterium and Dialister were more abundant in the controls.

### 3.6 Functional prediction based on KEGG pathways

**Figure 9:**
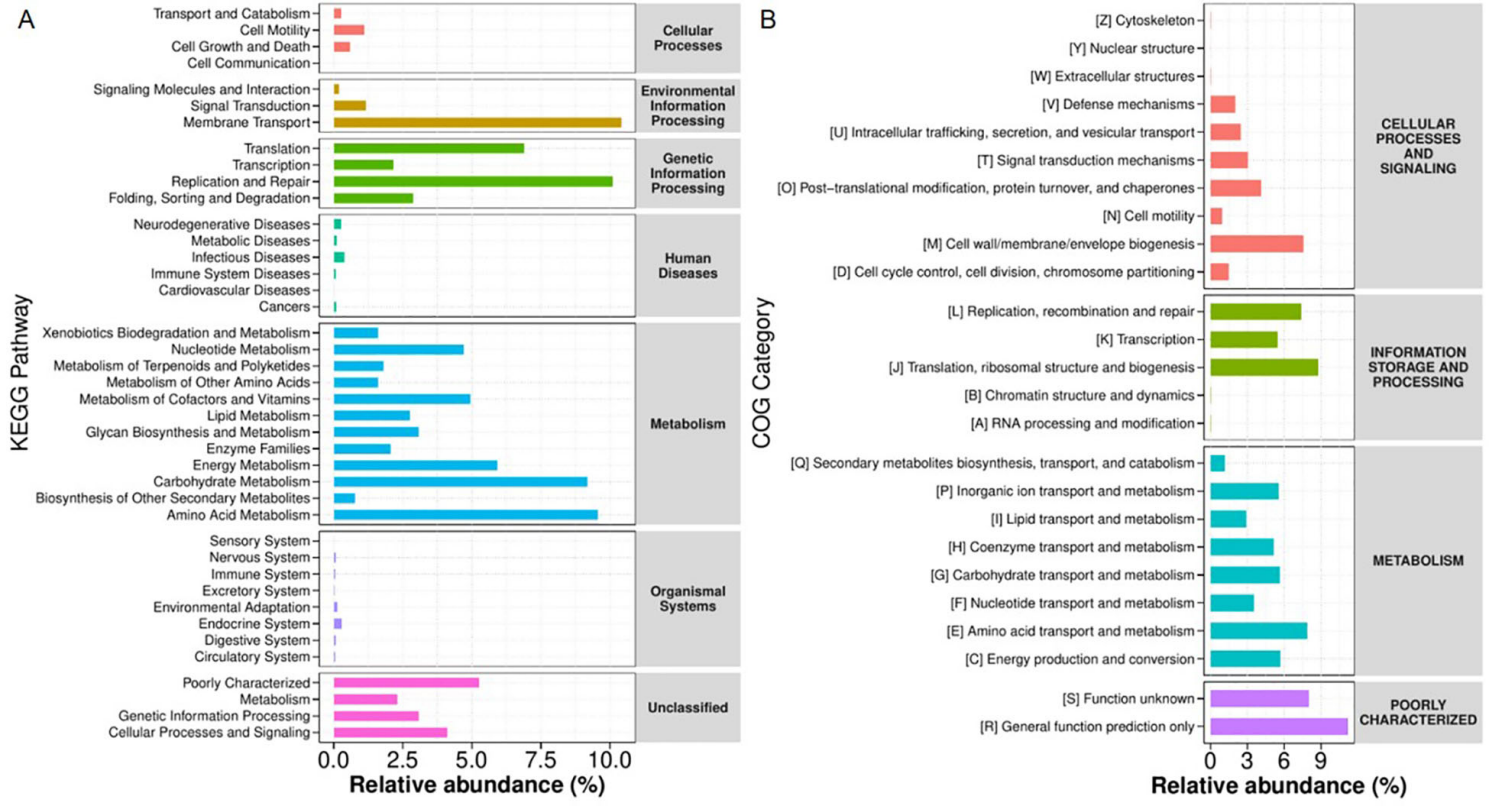
Predicted KEGG **(A)** and COG **(B)** pathways in the oral microbiota.

Functional profiling of the salivary microbiota based on KEGG (**Figure 9A**) and COG (**Figure 9B**) databases revealed a consistent dominance of core metabolic processes. The most abundant functions were associated with carbohydrate metabolism and amino acid metabolism, highlighting their central role in microbial survival in the salivary environment. Additionally, essential cellular processes, including translation, replication, repair, membrane transport, and signal transduction, were highly represented. These results underscore the metabolic versatility and functional adaptability of salivary microbial communities.

**Figure 10:**
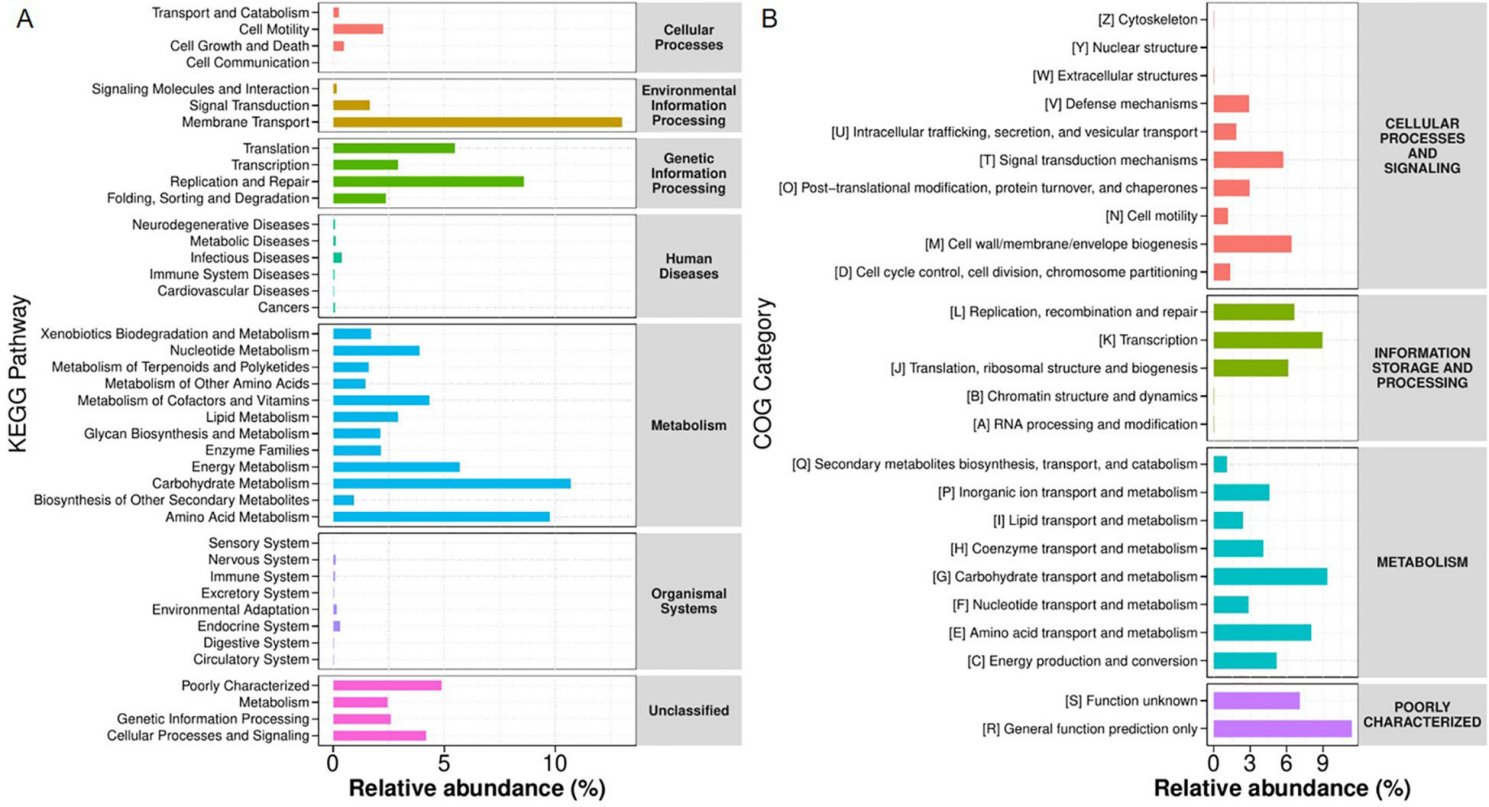
Predicted KEGG **(A)** and COG **(B)** pathways in the gut microbiota.

Functional analysis of gut microbiota demonstrated consistent metabolic dominance in both KEGG (**Figure 10A**) and COG (**Figure 10B**) databases. Carbohydrate and amino acid metabolism represented the most abundant pathways, accompanied by significant representation of membrane transport and genetic information processing.

## 4 Discussion

Our study characterized the oral and gut microbial communities in hemodialeysis patients, distinguishing between those with and without HFpEF and comparing them to CON. Despite the notable stability of alpha diversity, our findings reveal significant and site-specific alterations. The oral microbiome’s structure was significantly shifted by both hemodialysis and HFpEF, highlighted by the enrichment of Anaerocolumna in the HF group. In contrast, the gut microbiome structure was not further altered by heart failure, but all dialysis patients shared a profound dysbiosis, marked by the depletion of the protective genus Faecalibacterium.

A key observation was the comparable alpha diversity across all three groups for both oral and gut microbiota. This finding, which suggests similar community richness and evenness, contrasts with some reports of reduced microbial diversity in chronic kidney disease (CKD) (Vaziri et al., 2013; Saranya and Viswanathan, 2023). These discrepancies may be attributed to differences in diet, medication use, or dialysis duration. The oral microbiota’s stability may reflect limited exposure to uremic toxins compared to the gut. Notably, HF did not further reduce diversity, implying that dialysis treatment of uremia may dominate microbiome alterations.

Despite the stable alpha diversity, beta diversity analysis, reflecting overall community structural differences, revealed significant distinctions. For oral microbiota, clear clustering patterns indicated a substantial separation of the CON group from both hemodialysis groups (NHF and HFpEF), with further significant differentiation observed between the NHF and HF groups. This suggests a profound impact of ESRD and hemodialysis on oral microbial ecology, with additional, discernible alterations potentially attributable to heart failure pathology. In contrast, gut microbial communities (**Figure 3B**) did not exhibit statistically significant structural divergence among the CON, NHF, and HF groups. This suggests that, unlike the oral cavity, the presence of heart failure may not significantly alter the overall gut microbial community structure in hemodialysis patients. Our findings for oral beta diversity align with reports of systemic microbial shifts in chronic diseases (Altamura et al., 2023), including those in hemodialysis patients. However, the observed stability in gut community structure across our study groups provides a novel perspective, warranting further investigation into the specific factors that might stabilize the gut ecosystem in this particular cohort.

At the genus level, specific compositional shifts provided insight into this structural dysbiosis. In the oral cavity (**Figure 6A**), Veillonella, a commensal often involved in nitrate reduction beneficial for vascular health (Tonelli et al., 2023), was significantly depleted in HFpEF and NHF patients compared to CON. Conversely, genera such as Neisseria and Streptococcus became dominant in hemodialysis patients, suggesting a shift towards a community that is potentially more inflammatory or adaptive to the uremic oral environment (Duan et al., 2020; Xiang et al., 2024).

A finding was the significant enrichment of Anaerocolumna in the oral microbiome of the HEpEF group, positioning it as a notable candidate for further investigation. As a strict anaerobe from the Lachnospiraceae family (Ueki et al., 2016), its expansion in the oral cavity may reflect the more anaerobic and pro-inflammatory microenvironment characteristic of heart failure and uremia (Stenvinkel and Alvestrand, 2002). We hypothesize that Anaerocolumna is associated with systemic inflammation, which is closely related to the vicious circle of heart failure. However, its specific role is currently unclear. Our study identifies Anaerocolumna as a compelling candidate, the validation of which in larger, independent cohorts and through integrated metagenomic and metabolomic analyses will be essential to definitively clarify its potential mechanistic role.

Correspondingly, the gut microbiota (**Figure 6B**) displayed significant genus level alterations, with expansion of Bacteroides and Blautia in hemodialysis patients. More critically, the abundance of Faecalibacterium (Wang et al., 2020) was significantly reduced, as supported by findings from Li HB et al (Li et al., 2022). Its depletion exacerbates gut barrier dysfunction and uremic toxin accumulation, while supplementation via the butyrate-GPR43 axis attenuates renal inflammation. Previous studies have shown that ESRD patients exhibit significant enrichment of Bacteroides spp, which correlates with increased serum uremic toxins and impaired renal function. Bacteroides participates in aromatic amino acid (AAA) degradation, contributing to uremic toxin production. While Blautia is typically reduced in ESRD patients, its relative increase in certain subgroups may reflect compensatory metabolic adaptations to uremia. As a butyrate producer, Blautia is normally depleted in advanced CKD, but its expansion in HD patients could indicate diet- or dialysis-specific microbiome remodeling. Its depletion likely contributes to increased gut barrier permeability and the subsequent translocation of microbial products like endotoxins, a known factor in the pathogenesis of cardiovascular disease in CKD patients (Ramezani and Raj, 2014). The observed expansion of Bacteroides is also clinically relevant, as certain species within this genus are known to produce protein-bound uremic toxins, such as p-cresyl sulfate, from dietary amino acids (Kanjanabuch et al., 2009; Yang et al., 2018).

The distinct microbial signatures in the oral and gut cavities raise questions about their potential interplay via the oral-gut axis. The daily translocation of oral bacteria to the gastrointestinal tract is a physiological process, but it may become pathogenic in the context of a compromised gut. Oral pathobionts, such as Anaerocolumna enriched in our HFpEF patients, could find a niche for ectopic colonization in a gut environment depleted of protective taxa like Faecalibacterium. Such a mechanism, where oral bacteria are closely related to gut dysbiosis and systemic inflammation through migration., has been proposed in other inflammatory conditions (Kitamoto et al., 2020; Guo et al., 2024). While our cross-sectional data cannot confirm this directional link, it highlights a plausible mechanism for how oral dysbiosis could amplify systemic pathology in this vulnerable population.

This study has several limitations. Its cross-sectional design precludes any conclusions on causality. Due to the limitations of the retrospective design, data on smoking history, dietary habits, detailed medication history, and residual renal function were incomplete and could not be included in the analysis. The sample size, although controlled by PSM, is modest, while adequate for exploration, it prevented the development of a robust, covariate-adjusted prediction model or a meaningful cross-validated ROC analysis. Furthermore, the sample size of this exploratory study is modest (~30 per group), which limits its statistical power. This may have reduced our ability to detect more subtle microbial associations. Consequently, our findings represent candidate associations, not validated biomarkers, and warrant validation in larger cohorts using the suggested statistical frameworks. Our study identifies Anaerocolumna as a promising, non-invasive salivary microbiome associated with HFpEF in hemodialysis patients. However, this finding is derived from a single-center cohort with a limited sample size. To advance this discovery towards clinical utility, it is imperative to validate the robustness and generalizability of Anaerovoracaceae in large-scale, multi-center, and prospective studies that recruit participants from diverse ethnic and geographic backgrounds. In addition, future studies with larger, prospectively recruited cohorts must employ multivariate models to control for clinical confounders and confirm the independent effect of HFpEF identified here. And we did not collect standardized clinical oral health data, such as periodontal indices like probing depth, clinical attachment level, or caries indices. It is well-established that periodontal disease and oral hygiene practices are strong drivers of salivary microbiome composition and diversity. Consequently, we cannot rule out the possibility that the observed differences in the salivary microbiome are partially confounded by underlying, unmeasured variations in oral health status among the participants. Future studies designed to investigate the salivary microbiome in similar contexts should prioritize the inclusion of comprehensive oral clinical examinations as essential covariates to disentangle these complex relationships. Furthermore, this study was based on 16S rRNA gene sequencing, which provides taxonomic but not direct functional information. While inferences can be made based on the known metabolisms of differentially abundant taxa, such predictions are indirect and may not fully capture the complex functional dynamics *in situ*. To truly unravel the functional implications of the compositional shifts observed in our study, future research should integrate multi-omics strategies. As suggested by a growing body of literature, coupling 16S rRNA data with metagenomic sequencing can directly assess the genetic potential of the microbiome (Hu et al., 2025). Furthermore, a more direct and powerful approach would be to correlate taxonomic changes with microbial metabolic output through metabolomic analyses (Tintelnot et al., 2023). And applying this to our research context would be a logical and critical next step. Future research employing shotgun metagenomics and metabolomics is necessary to confirm the functional consequences of the observed dysbiosis and to elucidate the mechanistic links between specific microbes, their metabolic products, and the progression of HFpEF in hemodialysis patients. And we also should aim to culture target bacteria from matched oral and gut samples or utilize shotgun metagenomic sequencing for high-resolution, strain-level tracking of microbial movement between these sites.

In conclusion, our investigation reveals significant and nuanced alterations in oral and gut microbial structures in hemodialysis patients, which are further exacerbated by heart failure. While alpha diversity was stable, distinct beta diversity patterns and pivotal shifts in genera like Veillonella, Bacteroides, and Faecalibacterium underscore a profound microbial dysbiosis. These findings advance our understanding of the intricate interplay between the microbiome, renal disease, and heart failure, providing a foundation for developing microbiome-targeted diagnostics and therapies to improve outcomes in this vulnerable population.

## 5 Conclusion

This study reveals that maintenance hemodialysis and concomitant HFpEF are associated with distinct microbial signatures. Heart failure significantly alters the overall structure of the oral microbiome, positioning Anaerocolumna as a microbial characteristics of interest associated with HFpEF in this population, which provides a new hypothesis for understanding the microbial mechanism of HFpEF. In contrast, while the overall gut microbial structure was not significantly altered by the presence of HFpEF, a profound dysbiosis was still evident, marked by the loss of the protective genus Faecalibacterium in all dialysis patients. These findings highlight the oral microbiome’s potential as a non-invasive indicator for HFpEF in this vulnerable population and underscore the complex, site-specific nature of microbial dysbiosis.

## Data Availability

The data set presented in this study can be found in the online repository. The data presented in the study are deposited in the NCBI repository, accession number PRJNA1367528.
